# Dengue Virus Type 2 (DENV2)-Induced Oxidative Responses in Monocytes from Glucose-6-Phosphate Dehydrogenase (G6PD)-Deficient and G6PD Normal Subjects

**DOI:** 10.1371/journal.pntd.0002711

**Published:** 2014-03-13

**Authors:** Abdullah Ahmed Al-alimi, Syed A. Ali, Faisal Muti Al-Hassan, Fauziah Mohd Idris, Sin-Yeang Teow, Narazah Mohd Yusoff

**Affiliations:** 1 Advanced Medical and Dental Institute, Universiti Sains Malaysia, Bertam, Penang, Malaysia; 2 Department of Microbiology, School of Medical Sciences, Universiti Sains Malaysia, Kubang Kerian, Kelantan, Malaysia; Tropical Medicine Institute Pedro Kourí, Cuba

## Abstract

**Background:**

Dengue virus is endemic in peninsular Malaysia. The clinical manifestations vary depending on the incubation period of the virus as well as the immunity of the patients. Glucose-6-phosphate dehydrogenase (G6PD) deficiency is prevalent in Malaysia where the incidence is 3.2%. It has been noted that some G6PD-deficient individuals suffer from more severe clinical presentation of dengue infection. In this study, we aim to investigate the oxidative responses of DENV2-infected monocytes from G6PD-deficient individuals.

**Methodology:**

Monocytes from G6PD-deficient individuals were infected with DENV2 and infection rate, levels of oxidative species, nitric oxide (NO), superoxide anions (O_2_^−^), and oxidative stress were determined and compared with normal controls.

**Principal Findings:**

Monocytes from G6PD-deficient individuals exhibited significantly higher infection rates compared to normal controls. In an effort to explain the reason for this enhanced susceptibility, we investigated the production of NO and O_2_^−^ in the monocytes of individuals with G6PD deficiency compared with normal controls. We found that levels of NO and O_2_^−^ were significantly lower in the DENV-infected monocytes from G6PD-deficient individuals compared with normal controls. Furthermore, the overall oxidative stress in DENV-infected monocytes from G6PD-deficient individuals was significantly higher when compared to normal controls. Correlation studies between DENV-infected cells and oxidative state of monocytes further confirmed these findings.

**Conclusions/Significance:**

Altered redox state of DENV-infected monocytes from G6PD-deficient individuals appears to augment viral replication in these cells. DENV-infected G6PD-deficient individuals may contain higher viral titers, which may be significant in enhanced virus transmission. Furthermore, granulocyte dysfunction and higher viral loads in G6PD-deificient individuals may result in severe form of dengue infection.

## Author Summary

An estimated 50 to 100 million cases of dengue fever occur each year worldwide. Among these, there are 200,000 to 500,000 cases of life-threatening dengue hemorrhagic fever (DHF) and dengue shock syndrome (DSS). Factors contributing to the development of DHF/DSS are not yet fully identified. Glucose-6-phosphate dehydrogenase (G6PD) deficiency is prevalent in Southeast Asian countries where dengue is also endemic. Besides affecting normal function of erythrocytes, G6PD deficiency also affects other cells by causing abnormal cellular redox. Altered redox state of cells may render them less effective in clearing up microbial and viral infections. Here we confirm previous findings that monocytes from G6PD-deficinet individuals support better dengue virus replication. In addition, we show that reduced production of reactive oxygen, and nitrogen species and elevated levels of oxidative stress are responsible for the enhanced viral replication. We suggest that redox imbalance observed in infected monocytes from G6PD-deficient individuals may facilitate dengue transmission and affect clinical outcome. However, a handful of studies carried out in areas where both G6PD deficiency and dengue are endemic, reveal no statistically significant correlation between severity of Dengue and G6PD deficiency. Well-designed studies are needed to demonstrate that G6PD-deficient individuals are at risk of severe dengue.

## Introduction

Dengue infection is among the leading causes of morbidity and mortality in the tropics and subtropics where as many as 100 million people are infected with 22,000 deaths yearly [1]. Dengue infection is caused by dengue virus (DENV), an RNA virus of the family *Flaviviridae*. There are four serotypes of the virus which are referred to as DENV1, DENV2, DENV3 and DENV4. All four serotypes can cause the full spectrum of disease [2]. DENV is primarily transmitted to humans by the bite of infected *Aedes* mosquitoes, particularly *Aedes aegypti*. Other *Aedes* species that transmit the disease include *A. albopictus*, *A. polynesiensis*, and *A. scutellaris* [3]. Although uncommon, DENV can also be transmitted via infected blood products and through organ transplantation [4], [5].

A large percentage (∼80%) of people infected with DENV show only mild symptoms such as fever. On the other hand, some patients experience more severe illness such as dengue hemorrhagic fever (DHF) and dengue shock syndrome (DSS), which can be life threatening [6]. Several factors have been associated with the development of severe dengue including prior infection with a heterotypic serotype, the strain of infecting virus, age and gender, and the genetic background of the patient. DENV-related illness is more common in children, and in contrast to other infections, well-nourished children are more predisposed to severe illness [7]. Polymorphism in genes coding for tumor necrosis factor alpha (TNFα), mannan-binding lectin 2 (MBL2), Cytotoxic T-Lymphocyte Antigen 4 (CTLA4), transforming growth factor β (TGFβ), DC-SIGN, and human leukocyte antigen (HLA) class I and II alleles have been linked with an increased risk of severe dengue complications [8]. Yet another genetic abnormality reported to have a link with DHF/DSS is the deficiency of glucose-6-phosphate dehydrogenase (G6PD), a ubiquitous X-linked enzyme which is part of the innate defense mechanisms [9].

G6PD deficiency is the most common enzymopathy worldwide, with highest prevalence among Sub-Saharan African countries [10],[11]. High frequencies (6.0–10.8%) of G6PD deficiency have also been reported for Southeast Asian countries [12]. G6PD deficiency affects the production of reactive nitrogen (RNS) and oxygen species (ROS) such as nitric oxide (NO), superoxide (O_2_^−^), and hydrogen peroxide (H_2_O_2_) resulting in alterations of normal redox state of the cells [13]. Cells of immune system employ RNS/ROS to kill invading pathogens. A reduction of the redox state of immune cells may render immune cells less effective against invading organisms, resulting in an increased severity of the infection [14].

It is found that monocytes from G6PD-deficient patients show an increased susceptibility to DENV2 infection with higher replication ability than those from normal controls [9]. Although there appears to be a connection between G6PD deficiency and enhanced DENV replication [15], no studies have been carried out to elucidate the molecular mechanism behind this observation. In the present study, we aim to find out whether it is the altered redox state of monocytes from G6PD-deficient individuals, which is responsible for the increased susceptibility of monocytes to enhanced DENV replication.

## Materials and Methods

### Ethics statement

This study was approved by the research and ethics committee of Clinical Research Center (CRC), Ministry of Health and Research, and Ethics Committee, Universiti Sains Malaysia. The study was carried out between January 2010 and December 2011. Written informed consent was obtained from all the blood donors who agreed to participate in the study.

### Study subjects and blood samples collection

Standard inclusion criterion for blood donors was followed and only male donors were included. Additionally, donors with prior history of DENV infection were excluded. After blood was donated, a portion (2.5 mL) of blood from the tubing of the blood bag was taken. The blood was screened for G6PD deficiency using ultraviolet test (Cat #: SQMMR 500, R&D system, Athena-Greece) and G6PD activity was assayed using G6PD kit (Cat #: PD2616, RANDOX Laboratory Antrim, UK) following manufacturers' protocols. Donors without G6PD deficiency on screening were selected as controls. The sera of G6PD-deficient and G6PD-normal donors were screened for DENV-reactive antibodies using IgG/IgM Capture ELISA kits (IgM Cat #: E-DEN01M and IgG Cat #: E-DEN02G, Panbio, Brisbane, Australia) according to the manufacturer's protocol.

### Virus propagation and titration

The C6/36 mosquito *A. albopictus* cell line (CRL-1660 ATCC, USA) was used to propagate DENV2 (D2MY00-22563), kindly provided by Prof Shamala of University of Malaya, in Leibovitz culture medium (L-15) (SIGMA, USA) supplemented with 1% L-glutamine (SIGMA, USA), 19% tryptose phosphate broth (Hi MEDIA, India), 1% of penicillin/streptomycin (GIBCO, Grand Island, USA) and 5% fetal bovine serum FBS (GIBCO, Grand Island, USA). DENV2 in conditioned medium was titrated using a plaque assay on Vero cells (CCL-81 ATCC, USA) essentially as described previously [16] and stored at −80°C in aliquots.

### Isolation and purification of monocytes

Peripheral blood mononuclear cells (PBMCs) were isolated from both G6PD-deficient and normal controls' blood by density gradient centrifugation using Lymphoprep medium (Cat #: N07-1114547 Axis Shield, Norway) following manufacturer's protocol. PBMCs were then used to isolate primary monocytes using a MACS kit system II (Cat #: 130-091-153, MiltenyiBiotec GmbH, Gladbach, Germany). The purified monocytes were enumerated and cell viability was determined by trypan blue exclusion assay. The cells were then seeded in 24-well plates (Costar, Corning, USA) at 2×10^5^cells/well and maintained in humidified incubator at 37°C in the presence of 5% CO_2_.

### Ex-vivo infection of monocytes

Monocytes from G6PD-deficient and normal controls were infected with DENV2 at multiplicity of infection (MOI) 0.1 for 3 hours at 37°C/5% CO_2_ as described previously [9]. For optimal virus contact to the monocytes, the plates were gently agitated every 15 min. After 3 hours of incubation, the cells were washed twice with serum-free medium, re-suspended in complete growth medium and cultured at 37°C/5% of CO_2_ for five days. Mock-infected monocyte cultures were set simultaneously as negative controls. Conditioned media were harvested at various time points (24, 48, 72, 96, 120 hours) post-infection and the number of infected cells and virus titers were determined using flow cytometry and plaque assay respectively. Virus-containing conditioned media were stored in aliquots at −80°C.

### Intracellular detection of DENV2 by flow cytometry

Infected and mock-infected monocytes were harvested and washed twice with cold phosphate buffered saline (PBS) containing 0.1% NaN_3_ (SIGMA-USA). Surface labeling was performed by incubating the cells with 100 µl (1∶10 diluted) of CD14-PE MAb (Cat#: 347497 Becton Dickinson, San Jose, CA, United States) for 30 min on ice in dark. Cells were fixed and permeabilized in 200 µl Cytofix/Cytoperm solution (BD Biosciences, San Diego, CA) for 15 min at room temperature followed by 2× washing in Cytoperm/Cytowash solution. Cells were stained with 100 µl (1∶20 diluted) of DENV2 E protein-specific monoclonal antibody (Abcam # ab41349) and incubated for 1 hour on ice in dark and washed twice with Cytoperm/Cytowash solution. Secondary FITC-labeled goat anti-mouse-IgG (Cat #: 349031, Becton Dickinson, Biosciences USA) antibody was added to a final concentration of 3.5 µg/ml and incubated for 30 minutes on ice in dark. Finally, the cells were washed twice in Cytoperm/Cytowash solution and resuspended in 0.5 ml of 1% paraformaldehyde containing 0.1% NaN_3_ and subjected to flow cytometry. Mock-infected monocytes were used as negative controls and were treated and run simultaneously with the infected groups (G6PD-deficient and G6PD-normal monocytes) to improve gating, setup of compensation and the precision of measurement. A minimum of 10,000 events were acquired using a FACS Calibur II flow cytometer (Becton Dickinson, Biosciences, USA). Data were analyzed using flowing software Ver 1.0 (Turku Center for Biotechnology, University of Turku, Finland http://www.flowingsoftware.com/). Isotype-matched antibody was used as a negative control. The percentage of DENV2 positive cells was determined from FITC fluorescence histograms using a region that was defined based on the analysis of the mock-infected control cells.

### Extracellular determination of DENV2 by plaque assay

Dengue virus particles were titrated by plaque assay as previously described by Roehrig JT et al. [16]. Briefly, Vero cells were seeded in 12-well plates at a density of 2×10^5^ cells/well in complete DMEM medium (without phenol red) and incubated overnight at 37°C/5% CO_2_. Serial 10-fold dilutions of virus-containing supernatant in DMEM/2% FBS were added to the wells and incubated at 37°C/5% CO_2_ for 90 minutes with gentle agitation every 15 minutes. The medium was carefully removed and the cells were overlaid with 1.0 ml of DMEM/10% FBS containing 0.5% carboxymethylcellulose (CMC) (SIGMA - USA) and incubated at 37°C/5% CO_2_ for 7 days. CMC was removed by washing the wells twice with 2.0 ml PBS/well. Cells were fixed with freshly prepared cold 4% paraformaldehyde and stained with 1% crystal violet (AMRESCO, USA) in 20% ethanol for 30 minutes. The viral titers were expressed as plaque forming units (PFU)/ml = [(number of plaques per well)×(dilution)]/(inoculums volume).

### Detection of nitric oxide (NO) released from infected monocytes

Nitric oxide (NO) released into the conditioned media of DENV2 and mock-infected monocytes was determined by measuring the stable nitrite using a Greiss reagent assay kit (Cat #: R&D SYSTEM) according to the manufacturer's protocol. The absorbance was measured at 540 nm using Bio Mate 3 (Thermo Scientific, USA) spectrophotometer. All measurements were performed in duplicates.

### Detection of superoxide and oxidative stress in infected monocytes

The intracellular superoxide anion and ROS production were quantified in the DENV2 infected and control cells using a total ROS/Superoxide detection kit (Cat #: ENZ 51010, ENZO LIFE SCIENCES INC., USA) following manufacturer's protocol. Briefly, DENV2 and control cells were harvested at 24, 48, 72, 96, and 120 hours and washed using ROS buffer supplied with the kit. The positive, negative, DENV2-infected and control cells were then incubated with 500 µl ROS/superoxide detection reagents for 30 minutes at 37°C/5% of CO_2_ in RPMI-1640/10% FBS and subjected to flow cytometry analysis. Data analysis and anticipated results were obtained by generating a log FL1 (X-axis) versus a log FL2 (Y-axis) dot plot with quadrants added to it. Monocytes with increased production of superoxide demonstrated a bright orange fluorescence and detected using the FL2 channel appeared in the two upper quadrants of a log FL1 (X-axis) versus a log FL2 (Y-axis) dot plot. Monocytes with high production of oxidative stress demonstrated a bright green fluorescence and registered in FL1 channel appeared in the upper and lower right quadrants of a log FL1 (X-axis) versus a log FL2 (Y-axis) dot plots. Results of experiments are presented as a percentage of the cells with increased superoxide and ROS production or as an increase in the mean fluorescence of induced samples versus controls.

### Statistical analysis

Results were reported as mean ± SD. Data were analyzed using SPSS software version 11.5. The *p*-value was calculated using Student's T test. A *p*-value less than 0.05 were considered significant. Error bars were expressed as means ± SD.

## Results

### G6PD screening of subjects

Four hundred blood donors were screened for G6PD enzyme deficiency and 16 donors were found to be G6PD deficient by the ultraviolet test. Out of 384 G6PD-normal donors, 16 were matched for age (33.18±4.28 years for G6PD-normal and 34.95±4.34 years for G6PD-deficient) and selected as normal controls (G6PD-normal donors). The G6PD deficient (*n* = 16) and age-matched G6PD-normal donors (*n* = 16) were then subjected to G6PD enzyme activity assay. G6PD-normal donors will be referred to as normal controls from hereafter.

### G6PD enzyme activity of subjects

**Figure 1:**
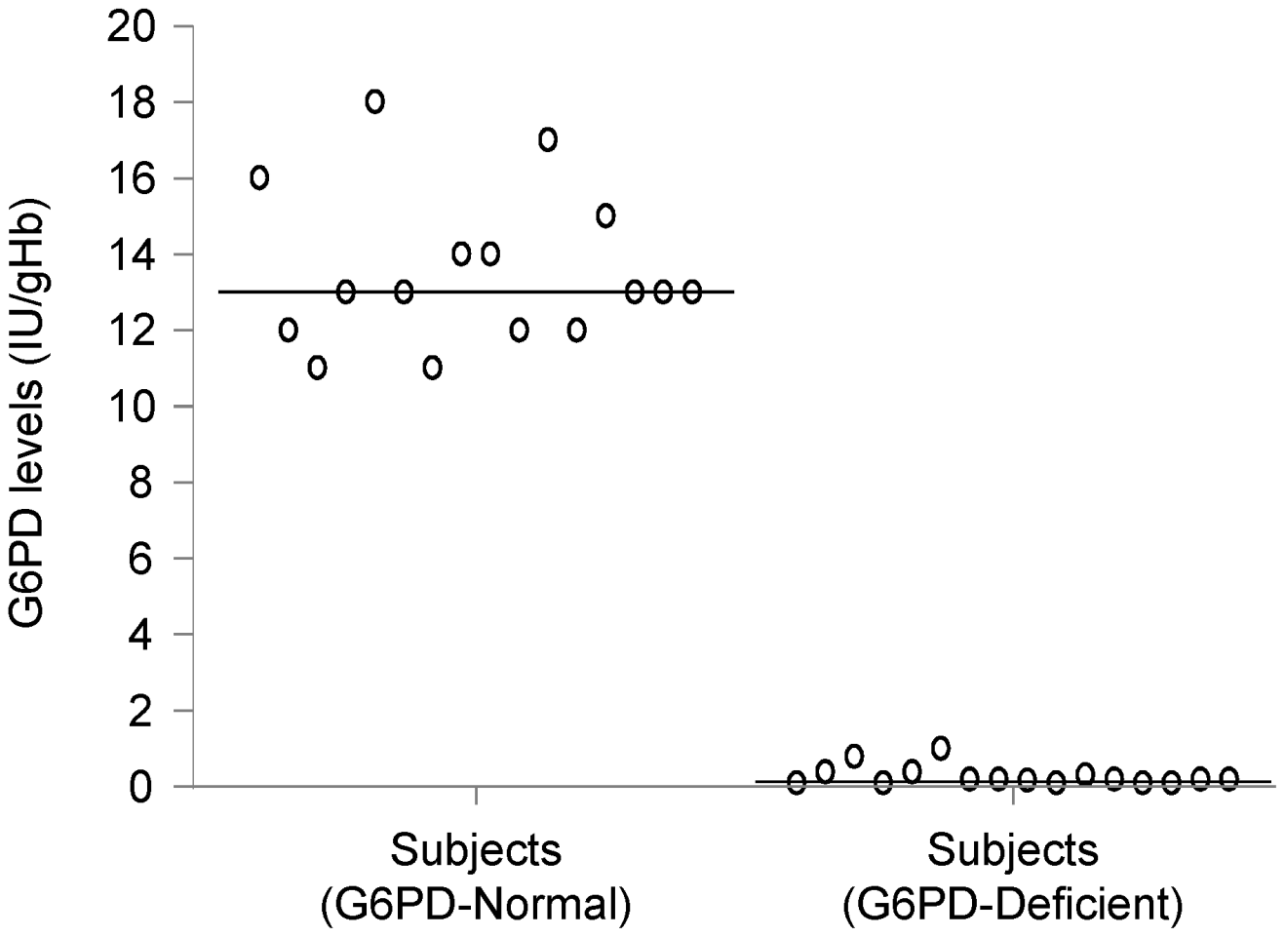
G6PD levels in normal controls and G6PD-deficient subjects. G6PD levels were determined by a commercially available fluorimetric assay. The mean G6PD activity for G6PD-deficient individuals was 0.285±0.26 IU/g Hb, compared to 13.56±2.02 IU/g Hb for age matched normal controls.

All 16 G6PD-deficient individuals had less than 10% of normal G6PD activity as measured by the fluorometric assay. The mean G6PD activity for G6PD-deficient individuals was 0.285±0.26 IU/g Hb, which was significantly (*p*<0.0001) lower than the mean activity of G6PD-age matched normal controls (13.56±2.02 IU/g Hb) as shown in **Fig. 1**.

### Growth curves of DENV2 in monocytes of G6PD-deficient and normal controls

**Figure 2:**
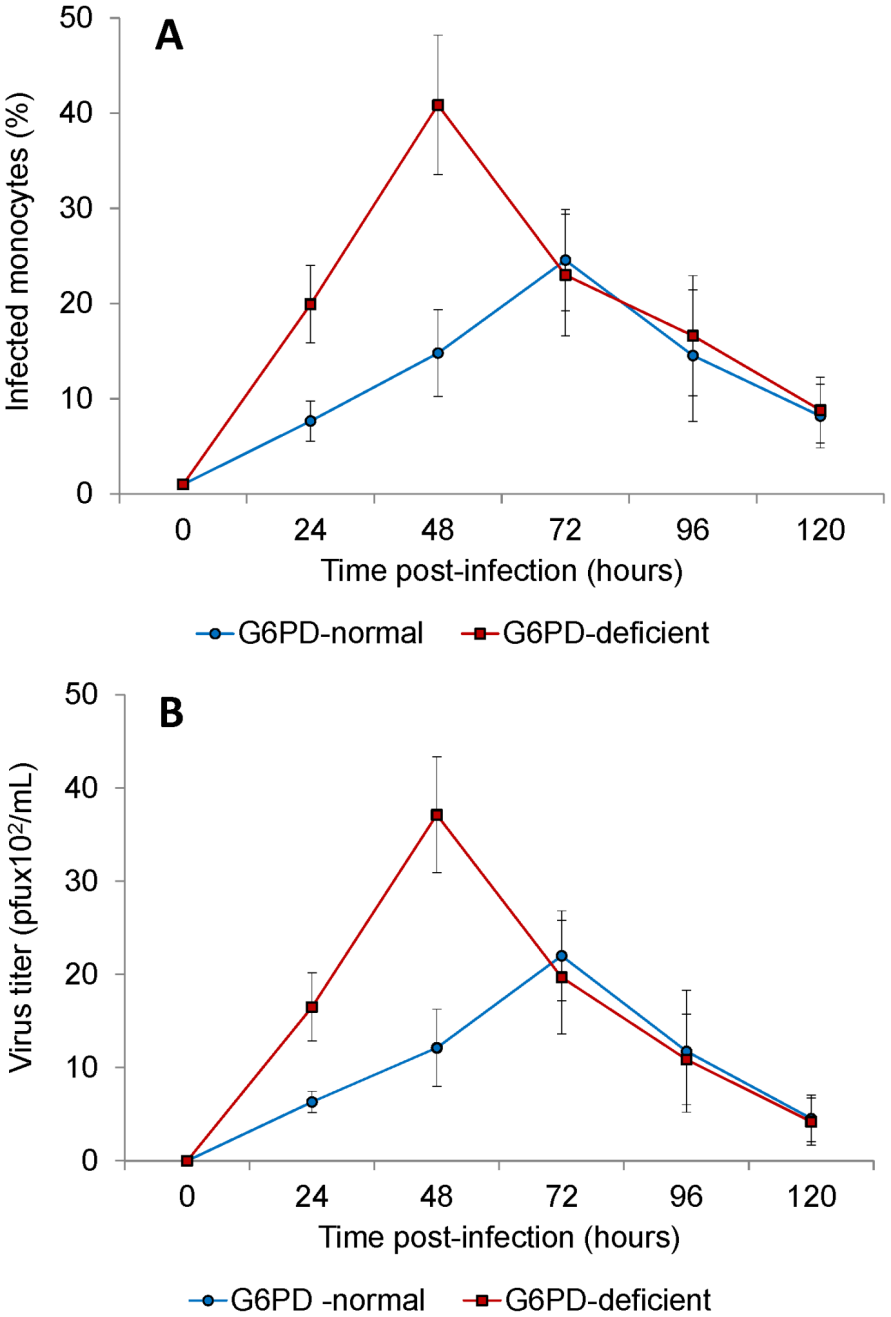
Growth curves of DENV2 in monocytes of G6PD-deficient and normal controls. The monocytes from G6PD-deficient and normal controls were infected with DENV2 at an MOI of 0.1. Cells and culture supernatants were harvested at 24, 48, 72, 96, 120 hours post-infection. Number of DENV infected cells were assayed by flow cytometry (**A**), whereas virus released by the infected cells was determined by Plaque assay (**B**). Number of infected cells as well as virus titer found to be significantly higher in infected monocytes from G6PD-deficient individuals compared to the normal controls.

In G6PD-deficient monocyte cultures, increased DENV2 infected cell rates were detected by flow cytometry until 48 hours post-infection, when peak values were reached. After this peak, the percentage of DENV2 infected cells decreased (**Fig. 2A**). Monocytes from 16 G6PD-deficient subjects showed mean virus infection percentage of 40.868±7.330% at 48 hours post infection. In normal control monocytes culture, the frequency of DENV2 infected cells increased until 72 hours post-infection, when peak values were reached followed by a decrease (**Fig. 2A**). Monocytes from 16 normal controls showed mean virus infection percentage of 24.3%±4.6% at 72 hours post infection. These results indicate that the mean percentage of DENV2-infected monocytes from G6PD-deficient subjects exceeded the percentage of DENV2-infected monocytes from normal controls. This difference of infected cells from two groups was statistically significant (p<0.0001). Moreover, the peak infection was delayed by 24 hours in monocytes from normal controls compared to monocytes from G6PD-deficient subjects.

This data was further verified by measuring the cell-free DENV2 in the supernatant of infected monocyte cultures (**Fig. 2B**). In G6PD-deficient monocyte cultures, increased DENV2 titers were detected by plaque assay until 48 hours post infection, when peak values were reached. After this time point, DENV2 titers decreased (**Fig. 2B**). DENV2 released from 16 G6PD-deficient monocyte cultures showed mean peak titers of 37×10^2^ PFU/ml on the 48 hours post infection. In normal control monocyte cultures, increased DENV2 titers were detected until 72 hours post infection, when peak values were reached. After this time point, DENV2 titers decreased (Fig. 1B). DENV2 released from 16 normal control monocyte cultures showed mean peak titers of 22×10^2^ PFU/ml on the 72 hours post infection.

These results indicate that significantly (*p*<0.0001) more DENV2 was released by the infected monocytes from G6PD-deficient subjects compared to infected monocytes from normal controls. The peak DENV2 titers were detected at an earlier time point (48 hours post infection) in G6PD-deficient monocyte cultures compared to normal control monocyte cultures (72 hours post infection).

### Nitric oxide (NO) production by monocytes of G6PD-deficient and normal controls

**Figure 3:**
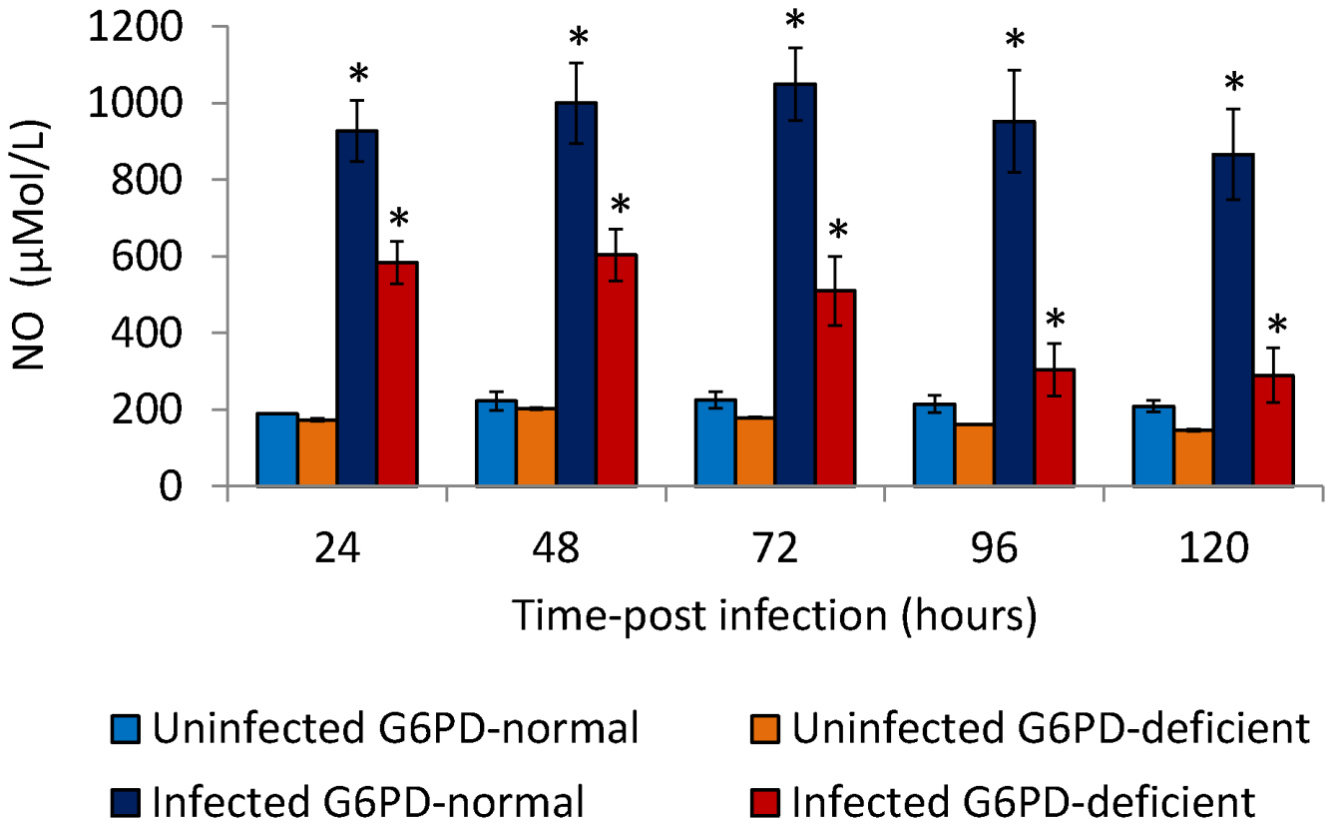
Production of nitric oxide (NO) in DENV-infected monocytes from G6PD-deficient, and normal controls. The production of nitric oxide (NO) in monocytes of both normal controls and G6PD-deficient donors increased significantly (*p*<0.001) after DENV2 infection. In a time-dependent manner, monocytes from G6PD-deficient subjects produced significantly (*p*<0.001) lower NO than monocytes from normal controls.

As illustrated in **Fig. 3**, DENV2 infection induced the production of NO in both normal control and G6PD-deficient monocytes in a time dependent manner. In G6PD-deficient monocyte cultures, increased NO produced until 48 hours post infection, when peak values were reached followed by a decrease (**Fig. 3**). NO released from 16 G6PD-deficient monocyte cultures showed mean peak levels of 603.75 µM L^−1^ at 48 hours post-infection. On the other hand, in the normal control monocytes, increased NO produced until 72 hours post-infection, when peak values were reached (**Fig. 3**). NO released from the normal control monocytes showed mean peak levels of 1050 µM L^−1^ at 72 hours post-infection. The difference in NO levels produced between G6PD-deficient and normal control monocytes was significant (*p*<0.0001) and the peak NO levels were detected at an earlier time point (48 hours post infection) in G6PD-deficient monocyte cultures compared to normal control monocyte cultures (72 hours post infection). The lower levels of endogenous NO production induced after the infection of G6PD-deficient monocytes correlate with the accelerated replication of DENV2 in these cells.

### Superoxide (O^2.−^) production by monocytes of G6PD-deficient and normal controls

**Figure 4:**
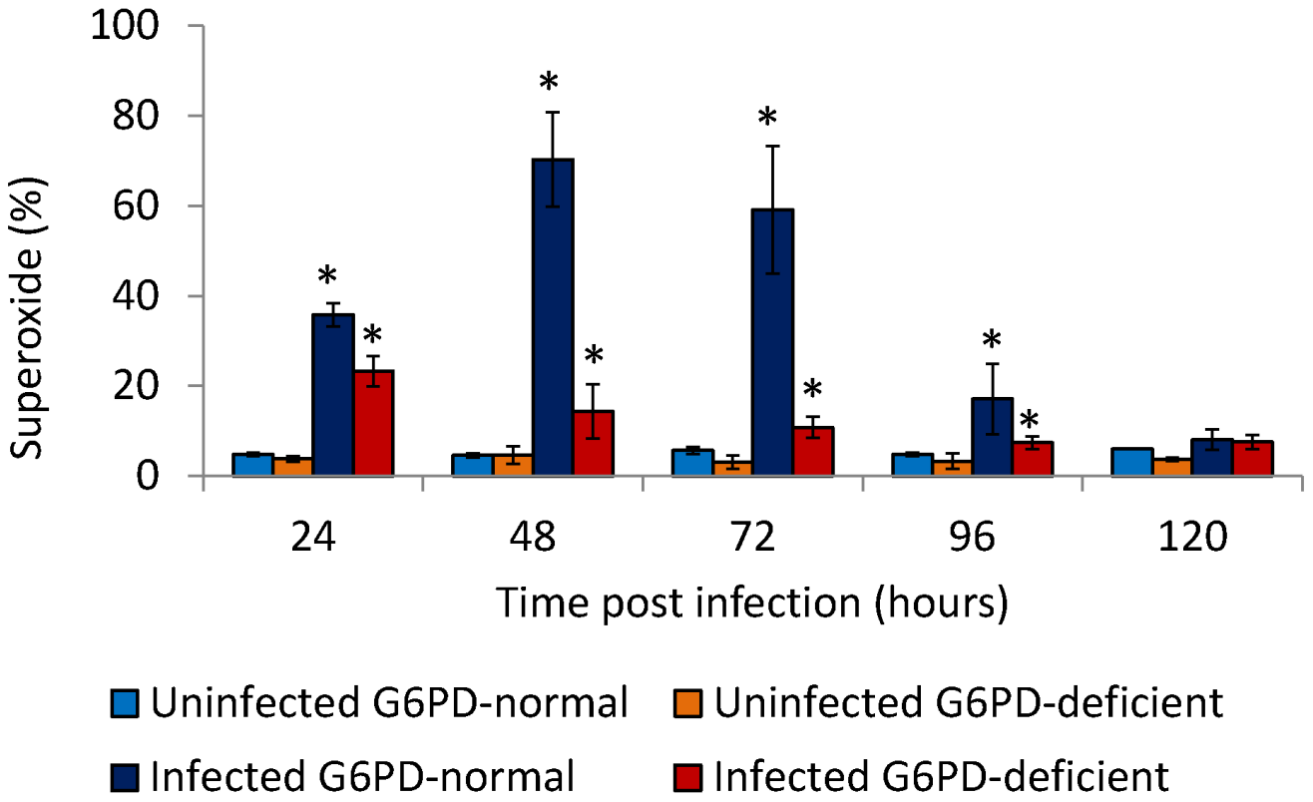
Production of superoxide anions (O^2.−^) in DENV-infected monocytes from G6PD-deficient, and normal controls. The production of superoxide anions (O^2.−^) in monocytes of both normal controls and G6PD-deficient donors increased significantly (p<0.001) after DENV2 infection. In a time-dependent manner, monocytes from G6PD-deficient subjects produced significantly (p<0.001) lower O^2.−^ than monocytes from normal controls.

As shown in **Fig. 4**, DENV2 infection of monocytes induced generation of superoxide anions (O^2.−^) in both normal control and G6PD-deficient monocytes (*p*<0.001) in a time dependent manner. In G6PD-deficient monocyte cultures, increased O^2.−^ levels were observed until 24 hours post infection, when peak values of 23.3% were reached (**Fig. 4**). On the other hand, in normal control monocyte culture, O_2_^.−^ levels increased until 48 hours post-infection, when peak value reached to 70.3% (**Fig. 4**). These results indicate that significantly (*p*<0.0001) less O_2_^.−^ was generated by the infected monocytes from G6PD-deficient subjects compared to infected normal control monocytes. The peak O_2_^.−^ levels were noticed at an earlier time point (24 hours post infection) in G6PD-deficient monocyte cultures compared to normal control monocyte cultures (48 hours post infection). The lower level of endogenous O_2_^.−^ generation induced after the infection of G6PD-deficient monocytes seems to markedly enhance the course of DENV2 infection in these cells.

### Oxidative stress in monocytes of G6PD-deficient and normal controls

**Figure 5:**
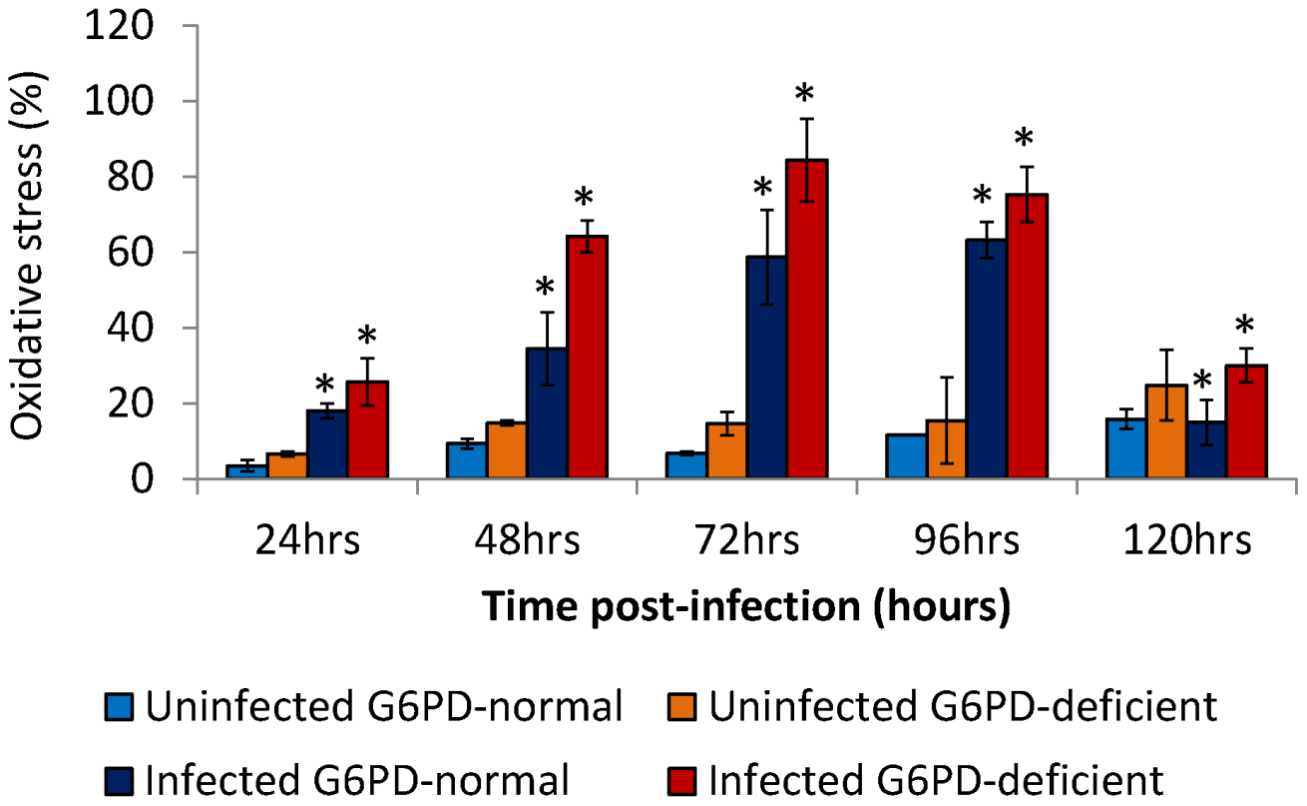
Oxidative stress accumulation in DENV-infected monocytes from G6PD-deficient and normal controls. The accumulation of oxidative stress in monocytes of both normal controls and G6PD-deficient donors increased significantly (p<0.001) after DENV2 infection. In a time-dependent manner, monocytes from G6PD-deficient subjects accumulated significantly (p<0.001) higher oxidative stress compared to monocytes from normal controls.

As displayed in **Fig. 5**, DENV2 infection of monocytes induced oxidative stress accumulation in both normal control and G6PD-deficient monocytes (*p*<0.001) in a time dependent manner. In G6PD-deficient monocyte cultures, oxidative stress accumulation increased until 72 hours post infection, when peak levels of 84.4% were reached (**Fig. 5**). Conversely, in normal control monocyte culture, oxidative stress accumulation increased until the 96 hours post-infection, when peak values of 63.2% were reached (**Fig. 5**). These results indicate that significantly (*p*<0.0001) more oxidative stress was accumulated by the infected monocytes from G6PD-deficient subjects compared to infected monocytes from normal control. The peak oxidative stress accumulation was noticed at an earlier time point (72 hours post infection) in G6PD-deficient monocyte cultures compared to normal control monocyte cultures (96 hours post infection). A higher and earlier accumulation of oxidative stress in G6PD-deficient monocytes appears to be a result of greater viral replication in these cells compared to normal control monocytes.

### Correlation of virus replication with NO, O_2_^.−^, and oxidative stress

**Figure 6:**
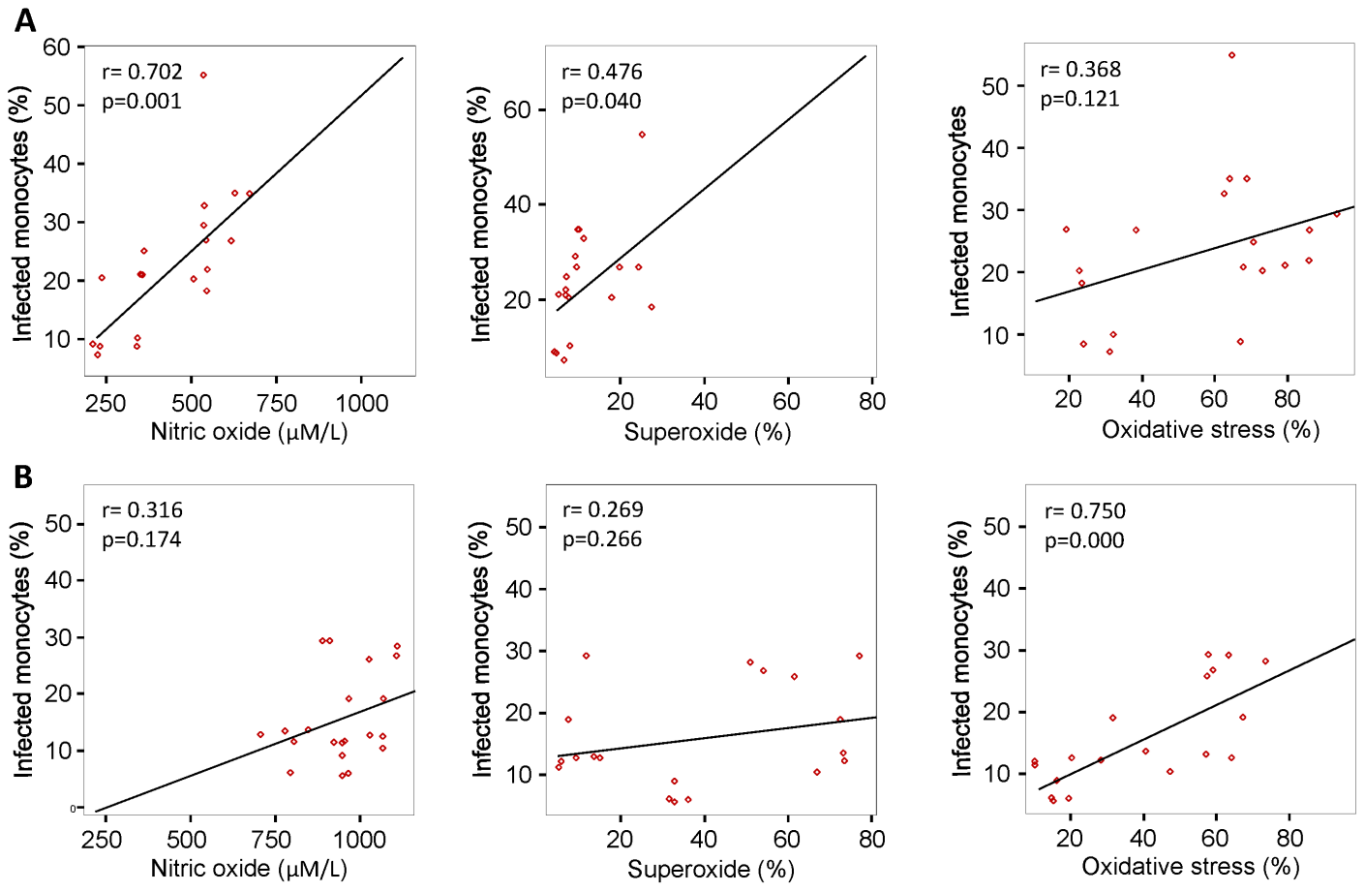
Correlation between DENV2 replication and NO, O^2.−^, and oxidative stress in monocytes from G6PD-deficient and normal controls. A significant moderate to strong correlation was found between the % infected monocytes and oxidative state (NO, O^2.−^, and oxidative stress) for monocytes from G6PD-deificient individuals (A) compared to normal controls (B).

There was a significant strong, positive correlation (r = 0.702; p = 0.001) between the DENV2-infected cells and NO levels in monocyte from G6PD-deficient subjects (**Fig. 6A**), compared to monocytes from normal controls (**Fig. 6B**). There was a significant moderate, positive correlation (r = 0.476; p = 0.040) between the DENV2-infected cells and O_2_^.−^ levels in monocyte from G6PD-deficient subjects (**Fig. 6A**), compared to monocytes from normal controls (**Fig. 6B**). There was also a significant moderate, positive correlation (r = 0.368; p = 0.121) between the DENV2-infected cells and O_2_^.−^ levels in monocyte from G6PD-deficient subjects (**Fig. 6A**), compared to monocytes from normal controls (**Fig. 6B**). These correlation studies further support the hypothesis that oxidative state of the cell may contribute to enhanced DENV infection.

## Discussion

DENV causes infection which may range from mild to severe in affected patients. The severe form is associated with DHF and DSS, which can be life threatening. The factors that define progression towards severe forms (DHF/DSS) of dengue infection remain to be elucidated. One possible correlate though may be the innate G6PD deficiency, which is the most common enzymopathy worldwide. G6PD, through the pentose phosphate pathway (PPP), provides the reduced form of NADPH for various cellular reactions including glutathione (GSH) recycling, superoxide anion production via NADPH oxidase, nitric oxide (NO) synthesis, and cholesterol synthesis [12].

Inhibition of G6PD results in the generation of less reactive oxygen and nitrogen species (super-oxide anion, hydrogen peroxide, hydroxyl radical, and NO) in the granulocytes and endothelial cells [17],[18]. Recurrence of microbial infections in G6PD deficient individuals has been reported [19]. The effect of cellular redox state on the course of viral infections is also well documented. For instance, replication of corona virus, coxsackie virus, rhinovirus, influenza, HIV, hepatitis, and enterovirus 71 virus is modulated by the redox milieu [20],[21],[22],[23],[24],[25]. Coxsackie viruses found replicate to a higher titer in C3H/JHe mice fed with diets deficient in selenium (Se), vitamin E or both than in mice given a normal diet [26]. Similarly, glutathione administration exhibited antiviral effect on influenza virus [22]. These findings suggest that redox imbalance may be conducive to enhanced replication and virulence of certain viruses.

Chao et al., [9] reported that monocytes from G6PD-deficient patients, using an ex vivo culture system, were more readily infected with the two DENV2 strains-(1) the New Guinea C strain from the DF patient or (2) the 16681 strain from the DHF patient than with those from normal controls. However, the underlying mechanism of this enhanced susceptibility was not investigated. Here we confirm Chao's findings and show in addition that reduced production of NO and O_2_^−^ as well as earlier accumulation of oxidative stress contribute significantly to enhanced DENV2 infectivity in monocytes from G6PD-deficient subjects.

Enhanced infection of G6PD-deficient monocytes by DENV may be attributable to increased viral receptors on these cells or greater production of viral particles or a combination of both. We have not studied the up-regulation of viral receptors but did clearly see an enhanced production of viral particles in these cells. Based on our findings and those reported by others, we propose a model for the association between the redox status of the host cells and DENV. Following entry into the cell, DENV fuses with endosomal membrane to release its nucleocapsid into the cytoplasm where viral RNA is replicated and translated into proteins. These processes may be affected by the cellular redox state, being more efficient in an oxidizing environment, thus resulting in enhanced virus production.

Findings of Chao et al., [9] and the ones presented here suggest that the high competency for DENV infection in monocytes of G6PD-deficient individuals may result in increased replication and higher virus yield. Higher viral loads in G6PD-deficient individuals may increase the probability of dengue transmission to others via infected mosquitos. Several reports have shown a correlation between severe dengue and high viral loads [27],[28], [29], [30], [31]. G6PD deficiency is reported to cause granulocyte dysfunction [18], [32]. A combination of granulocyte dysfunction and enhanced replication of DENV in monocytes of G6PD-deficient individuals may prevent the clearing of the primary infection thus predisposing infected individuals to severe form of disease.

It remains to be seen whether G6PD deficiency-mediated enhanced viral replication has any outcome on severity of dengue infection in areas where both dengue infection and G6PD deficiency are endemic. In Thailand, the prevalence of G6PD deficiency in the general population is approximately 14%. A cohort of 89 males diagnosed with DHF was studied to determine if G6PD deficiency was related to occurrence and/or course of dengue infection. Out of 89, a total of 17 (19.1%) DHF patients had G6PD deficiency, thus no significant association established between G6PD deficiency and DHF [15]. However, this study is based on only a modest number of hospitalized patients and therefore provides little information on the incidence of severe dengue in G6PD deficient individuals. High prevalence of G6PD is also reported in African population [33] but a low incidence of severe dengue is noted in populations of African origin in a couple of studies conducted in Cuba [34] and Haiti [35]. Since the outcome of these studies is inconclusive, well-designed studies are needed to demonstrate whether G6PD-deficient individuals are at risk of severe dengue with statistical significance. Such studies would be potentially beneficial in providing added knowledge of host defense mechanism, and may be clinically important for G6PD-deficient individuals travelling to or living in DENV endemic areas.
